# The strengths of the genetic approach to understanding neural systems development and function: Ray Guillery's synthesis

**DOI:** 10.1111/ejn.13985

**Published:** 2018-08-01

**Authors:** Anthony‐Samuel LaMantia

**Affiliations:** ^1^ Institute for Neuroscience and Department of Anatomy and Cell Biology The George Washington University School of Medicine and Health Sciences Washington District of Columbia

**Keywords:** developmental neurobiology, gene disruption in mice, neural differentiation, olfactory nervous system, visual system

## Abstract

The organization and function of sensory systems, especially the mammalian visual system, has been the focus of philosophers and scientists for centuries—from Descartes and Newton onward. Nevertheless, the utility of understanding development and its genetic foundations for deeper insight into neural function has been debated: Do you need to know how something is assembled—a car, for example—to understand how it works or how to use it—to turn on the ignition and drive? This review addresses this issue for sensory pathways. The pioneering work of the late Rainer W. (Ray) Guillery provides an unequivocal answer to this central question: Using genetics for mechanistic exploration of sensory system development yields essential knowledge of organization and function. Ray truly built the foundation for this now accepted tenet of modern neuroscience. His work on the development and reorganization of visual pathways in albino mammals—all with primary genetic mutations in genes for pigmentation—defined the genetic approach to neural systems development, function and plasticity. The work that followed his lead in a variety of sensory systems, including my own work in the developing olfactory system, proceeds directly from Ray's fundamental contributions.

## An Exemplary Mentor: My Training with Ray

Ray Guillery had an enormous influence on the scientific careers of the first, second and third generation of those we now call “neuroscientists.” My deep respect, affection and boundless gratitude reflect his remarkable capacity to encourage those in whom he believed. Ray's impact on the trajectory of my career, and indeed my life, was singular and essential. As a University of Chicago sophomore, I was the most unlikely candidate for a career in neuroscience that anyone could have imagined; anyone, that is, except for Ray. My interests did not include neurobiology, or developmental biology or any biology for that matter. Instead, I was passionate about literature and the arts. My arrival in Ray's lab at Chicago was due to this interest, as well as a glimmer of curiosity established by a course I had taken on the Brain and Behavior at the beginning of my sophomore year. The text for the course “Programs of the Brain” (Young, 1978) was by Ray's doctoral mentor J.Z. Young, although I had no idea at the time. I wrote my final paper on what I thought was “really interesting stuff” by some guys at Harvard, Hubel and Wiesel, on nerve cells in the brains of cats that “saw” specific edges and angles. I remember reading some of their papers in the reading room in Regenstein Library on the U. of C. Hyde Park campus, fascinated by it all, and not quite sure what to make of this very different way of thinking about the world and how we know it.

Ray's ability to help each individual in his laboratory finds his or her own place in the scientific ecosystem was one of his many essential contributions—the list of his collaborators and trainees who have gone on to successful careers in neuroscience is unsurpassed. Nevertheless, Ray's impact on neuroscience is best remembered through his remarkable scientific accomplishments—particularly his synthesis of genetics, neuroanatomy, physiology and developmental biology to generate a comprehensive understanding of the mammalian visual system. Ray's foundational influence on my career can be seen in my reliance on the integration of genetics and neuroanatomy to understand neural systems development. His work on retino‐geniculo‐cortical development, its disruption by albino mutations and the consequences of those genetic “lesions” for the assembly of a functional system provided a template for my own work on olfactory system development (reviewed in Balmer & LaMantia, 2011; LaMantia, 1999, 2014). This template, established first by Ray for the visual system, defined the essential role of sensory afferent interactions with their targets. This was somewhat at odds with the notion of target regulation of motor system development suggested by Viktor Hamburger and his colleagues (Hamburger, 1975; Hollyday & Hamburger, 1976; Purves, Snider, & Voyvodic, 1988). Ray's emphasis on sensory afferent interactions with their central targets in the developing visual system spurred new and fundamental understanding of somatosensory development, especially in rodent whisker “barrel” fields (Belford & Killackey, 1979; Killackey & Belford, 1979; reviewed by Erzurumlu & Gaspar, 2012) and primary auditory pathway development in a number of vertebrates (Born & Rubel, 1985; reviewed by Rubel, Hyson, & Durham, 1990). The work in my laboratory on mouse olfactory pathway development, reviewed here, was profoundly influenced by Ray's formulation of afferent influences on sensory system development and the value of genetics to understand those influences. The current concept of afferent/target interactions in shaping connectivity in all sensory systems thus traces back to Ray's work on albino mutants and his interpretations of the consequences of misrouting of retinal axons at the optic chiasm for visual system organization and function.

## Learning Neuroscience in the Guillery Lab

I joined Ray's lab in the summer of 1980, with little more than a bit of curiosity stimulated by Young, Hubel and Wiesel, and a lot of cluelessness. My arrival was due to coincidence rather than intention. Thanks to the suggestion of a friend with whom I had worked with at U of C's Court Studio Theater, I filled a summer job in Ray's lab that a Stanford undergraduate had declined at the last minute. This coincidence sets me on journey that continued, in large measure due to Ray's encouragement and support, from that Chicago summer until now. I spent the summer of 1980 in Ray's lab cutting, mounting and staining paraffin sections of the early postnatal ferret brain, and chirping a few questions (OK, more than a few). As summer turned to fall, Ray suggested that I should learn more about what was going on in the laboratory. We embarked on a series of “reading courses” starting in the autumn of my junior year. For the first quarter, we began with chapters by Lorente de No (1949), Mountcastle (1978) and Peters, Palay, and Webster (1976) on cortical anatomy, physiology and synaptic organization. In retrospect, I now appreciate that Ray's intention was for me to learn the importance of integrating careful, thorough anatomical analysis with physiological function. He knew that I had made a foray into understanding Hubel and Wiesel's work, and with this new information, he gave me a framework for understanding that work more clearly.

For the second quarter, we focused on how genetic mutations could alter cortical and subcortical organization. Ray had me read then “recent,” now “classic” papers on the *weaver* and *reeler* mutations in mice by Rakic (1976) as well as Stanfield and Cowan (1979). These papers focused on histogenesis in monogenic mutant mice and demonstrated a relationship between single genes, neurogenesis and cell migration in the cerebellum and hippocampus. Perhaps due to the then (and still) mysterious function of the brain regions analyzed in these mice, these studies had a primarily cell biological emphasis. They could not fully address how mutation disrupted the development of a functional “neural system” from its central nervous system source to its peripheral target or from its afferent origin to final destination. In other words, why did the *Weaver* weave, or the *Reeler* reel? The most striking readings, however, were those that I carried home from Chicago to Cleveland to absorb over the December holiday break. I sat in the library at Case Western Reserve University (a quiet spot, away from holiday distractions at home) and spent the better part of a day reading one single paper: “Abnormal retino‐geniculate and geniculo‐cortical pathways in several genetically distinct color phases of the mink” (Guillery, Oberdorfer, & Murphy, 1979). That day in Cleveland changed my life. Very few, particularly very few in Cleveland, can say that.

**Figure 1:**
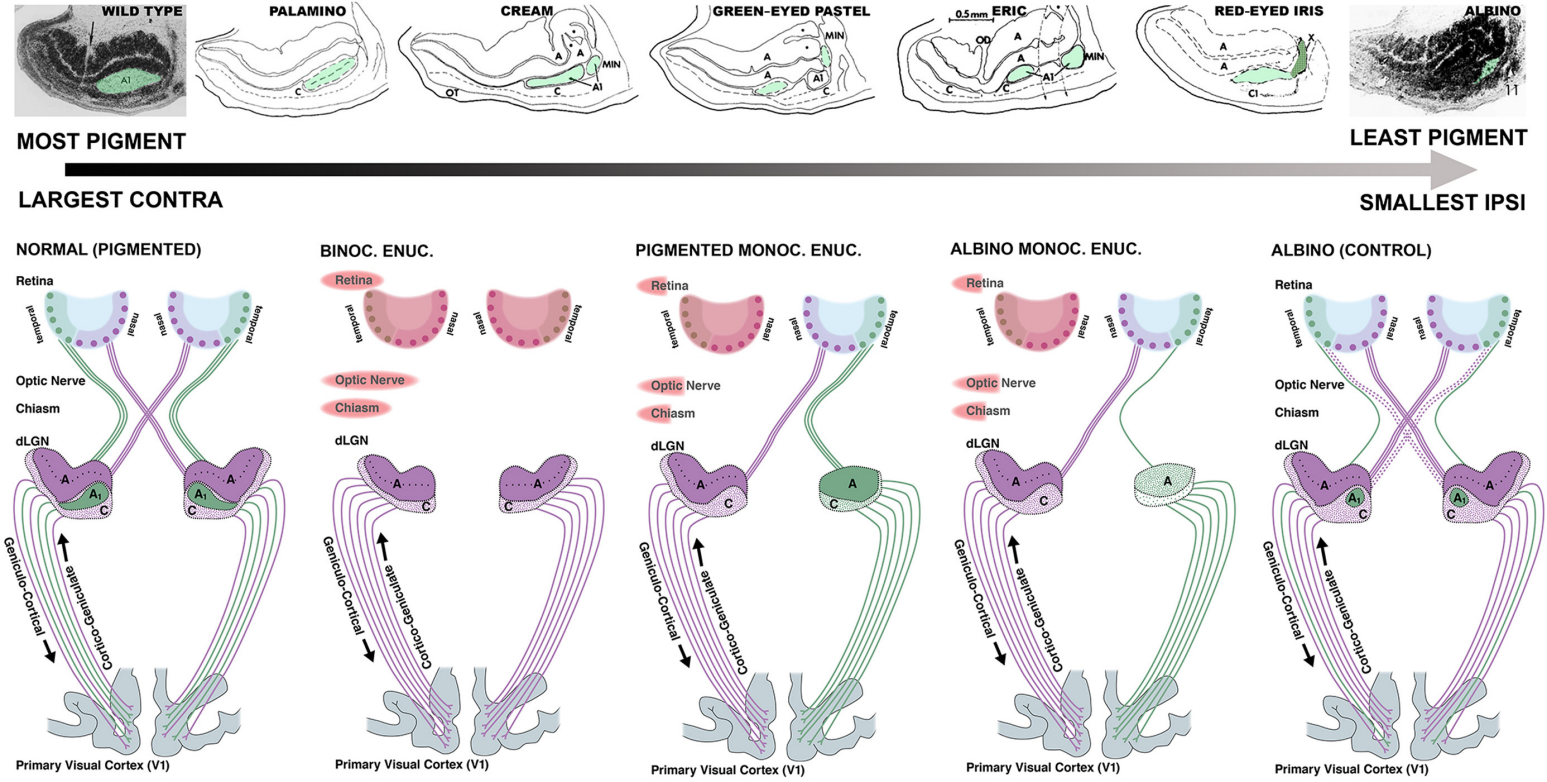
Consequences of genetic pigment variation and embryological change in ratios of contralateral versus ipsilateral retinal input for dorsal lateral geniculate nucleus (dLGN) lamination. Top row: The proportion and distribution of contralateral (targeting dLGN lamina A) versus ipsilateral (targeting dLGN lamina A1) retinal projections to the mink dLGN is related to differing levels of coat color pigment that parallel diminishing levels in the retinal pigment epithelium. The summary drawings of distinct color phases of mink (palamino, green‐eyed pastel, etc.) are presented in the order described by Guillery et al. (1979). The ipsilateral projection in each is highlighted in green to indicate the stepwise decrease in ipsilateral projections in the allelic series. In the red‐eyed iris mink, there is an ipsilaterally innervated region referred to as “X” (green hatched area) not considered part of the dLGN. Bottom row: An “embryological” series in pigmented and albino ferrets. dLGN retinal inputs have been altered by *in utero* or early postnatal binocular or monocular enucleation. dLGN development occurs with normal contra vs. ipsi innervation (*far left*) without any retinal innervation (binoc. enucleation; *middle left*), or differing proportions of contralateral and ipsilateral retinal input due to monocular enucleation in normally pigmented or albino ferrets (middle), with the smallest ipsilateral proportion present in the pigmented, nonenucleated albino ferret (far right). Top row from (Guillery et al., 1979); Bottom row based on (Guillery, LaMantia et al., 1985; Guillery, Ombrellaro et al., 1985).

What I learned from that paper, and in the subsequent examples that Ray had chosen for me, is that genetic variants, or mutations, provide perhaps the most incisive “natural experiment” possible to generate insight into how brain pathways (what we now, fashionably, call “circuits” and “systems”) develop, and ultimately how they work. The systematic variation in crossed and uncrossed retinal projections, geniculate lamination and geniculo‐cortical projections seen in each genetic “strain” or “color phase” of mink (carefully bred for their coat color by the fur industry) defined a central idea that has guided my entire career: Specific genes, in varying dosage, influenced by enhancers and suppressors, systematically account for the development and organization of specific brain structures and pathways. The conceptual advance wrought by Ray's (accompanied by co‐authors Mike Oberdorfer and E.H. Murphy) thorough and elegant description of the various color phases of mink was essential, and had a name that I would only learn subsequently: an “allelic series” (Figure 1, top row). This was most likely the first allelic series ever published that addressed how neuronal connections and maps are made.

In this remarkable study, Ray and his colleagues showed that the development, organization and function of an entire system, the mammalian visual system, could vary based upon different mutations in genes that account for quantitative distinctions in a single trait (the definition of an allelic series): in this case, pigmentation. He correctly determined that chiasmatic crossing of retinal axons was mediated by pigment‐related guidance signals. He showed clearly that the quantitative proportions and molecular identities of the contralateral and ipsilateral retinal afferents, when inappropriately versus appropriately targeted, had effects that concatenated throughout the visual system. The neurodevelopmental consequence of these mutations was surprising: The proportion of retino‐geniculate axons that cross versus those that do not cross at the optic chiasm varied in parallel with the degree of pigmentation in each mutant. The developmental response in the visual system was even more remarkable: The lamination of the lateral geniculate nucleus and the organization of its projections to the cortex were altered in direct proportion to the chiasmatic abnormalities. There was no cloning involved, no transcriptomics or any other “omics”; just careful classical genetics and elegant neuroanatomy.

The work that I did with Ray (with a major assist from John Robson) tested predictions generated from Ray's studies in mice (Guillery, Scott, Cattanach, & Deol, 1973), mink (Sanderson, Guillery, & Shackelford, 1974), ferrets (Guillery, 1971) and humans (Guillery, Okoro, & Witkop, 1975): all based upon the original work in Siamese cats (Guillery, 1969; Guillery & Kass, 1971). We evaluated the differentiation capacity of the early developing dorsal lateral geniculate nucleus (dLGN) in the absence of retinal inputs versus changes in the extent of retino‐geniculate innervation (Guillery, LaMantia, Robson, & Huang, 1985). Ray and I, working in ferrets (in which nearly 70% of the retino‐geniculate axons cross—providing a clear contra/ipsi distinction), and John working in mink (a mustelid with a similar retina‐geniculate pathway, see above, and Figure 1), manipulated the quantity of retinal input via binocular and monocular eye removal. We then assessed dLGN cytology, lamination and the pattern of remaining retino‐geniculate projections in animals in which retinal projections remained in normal and albino animals. The most extreme manipulation, bilateral eye removal in utero, prior to the arrival of retinal afferents in the dLGN, showed that the dLGN remained and was of similar size in pigmented or albino ferrets. Nevertheless, the developmentally deafferented nucleus was neither laminated nor were nuclear boundaries well differentiated. These dLGN rudiments, however, were bilaterally symmetrical, appropriately positioned in the dorsolateral thalamus, of reasonable size, and had some indication of “A” versus “C” laminae. In contrast, when one eye was removed at the same prenatal stage in pigmented ferrets or mink, the contralateral versus ipsilateral dLGN differentiated asymmetrically, in proportion to the remaining retinal input (Figure 1, bottom row). This asymmetry becomes accentuated, in register with further shifted proportions of contralateral versus ipsilateral projections, in monocularly enucleated albino ferrets (Guillery, LaMantia et al., 1985).

These experiments amplified conclusions from the allelic series in mink: dLGN development, when retinal input was present, adjusted proportionately to quantitative distinctions between contralateral versus ipsilateral afferents. In the complete absence of retinal input, however, the dLGN remained in the appropriate position; there was bilateral symmetry; the nucleus acquired a reasonable size; however, it had little cytological differentiation (Figure 1, bottom row). Ray, Mark Ombrellaro and I (Guillery, Ombrellaro, & LaMantia, 1985) went on to show that the remaining, still topographic geniculo‐cortical and cortico‐geniculate projections likely contribute to the preservation of rudimentary dLGN differentiation in the complete absence of retinal input. Indeed, it seemed possible—as suggested by Ray for the mink allelic series (Guillery et al., 1979)—that the quantitative mismatch of retino‐geniculate, geniculo‐cortical and cortico‐geniculate topographic inputs might underlie the adjustments in dLGN size and organization in a number of albino mutants (Guillery, Ombrellaro et al., 1985)—driving asymmetry in proportion with retinal afferents when present, and permitting symmetrical differentiation, albeit without several WT features, when retinal afferents were completely eliminated. For me, this was a first, essential lesson in how to analyze and interpret divergent development in wild type, mutant and experimentally manipulated animals. The pigment mutations themselves, though important, were not the primary focus, and the fetal manipulations were not “the main event.” The critical data were discerned based upon departures from typical neural pathway and systems development that were elicited by mutation or parallel experimental manipulation: Their integration and interpretation yielded mechanistic understanding of how a neural system is assembled.

Subsequently, Ray added another intriguing player into the cellular interactions necessary to sort the geniculo‐cortical and cortico‐geniculate projections by class and by topography: the cells of the thalamic reticular and peri‐reticular nucleus (Mitrofanis & Guillery, 1993). Ray and John M. suggested that the reticular and peri‐reticular cells might act in a way parallel to those in the cortical subplate (Ghosh, Antonini, McConnell, & Shatz, 1990; Kim, Shatz, & McConnell, 1991; McConnell, Ghosh, & Shatz, 1989) to segregate and direct the dLGN afferents to the cortex and cortical afferents to the dLGN. Such interactions, established independently, but influenced by, retino‐geniculate afferents (Grant, Hoerder‐Suabedissen, & Molnár, 2016) provide a mechanism for maintaining geniculo‐cortical/cortico‐geniculate topography in the extreme case of binocular enucleation, and in the reorganization of topography that results from quantitative changes in the proportion and mapping of ipsilateral versus contralateral retinal inputs in pigmented mutations in the mink and ferret. In addition, these observations provided insight into the cell biology of the maintenance of glomerular synaptic complexes in the dLGN despite the absence of retinal inputs. The developmental influence of the thalamic reticular nucleus secured the arrival of an appropriate set of cortical layer 4 through 6 terminals to form connections with dLGN cells. These dLGN cells, even in the absence of retinal input, retained instructions for forming specific synaptic architecture, and when confronted by the available cortical afferents, they did so. To paraphrase Ray, this mechanism, when operating in an intact retino‐geniculo‐cortical pathway, “allows retinal projections to plug into circuits that are to some extent already partially formed.” Beginning with the conundrum of retino‐geniculo‐cortical remapping in Siamese cats and other albino mutants, Ray and his colleagues had formulated an integrated cellular account of the mechanism by which retinal maps, thalamic relay and processing circuits and cortical representations were established.

In the summer of 1981, prior to beginning my senior year in college and my second year in Ray's lab, a review appeared in *Annual Reviews of Neuroscience* called “The Strengths and Weaknesses of the Genetic Approach to the Development of the Nervous System” by Gunther Stent, a pioneer molecular biologist, historian and philosopher of science (Stent, 1981). Stent shifted his attention in the late 1970s to understanding the organization and development of the nervous system. The variable cell lineages that Stent and colleagues demonstrated in the developing leech nervous system were compelling; however, the apparent indeterminate nature of these lineages for neuronal fate and connections led Stent to reject much of what he believed to be an excessively deterministic approach to understanding the assembly of neural systems and the use of genetics in this effort. The paper made quite a stir among the ranks of the Guillery lab at the time (including Chris Walsh, Linda Ide, Fernando Torrealba, Josephine Cucciaro, Virginia Holcombe and visiting professors Ulf Eysel and the late Ed Polley) because of its focus on whether genetics—particularly single gene mutations in a variety of species including Drosophila, mouse, and, of course, the Siamese cat—could be a useful tool in understanding how the nervous system is generated, and how connections are made. The animal that Stent chose for his own lineage studies, the leech, was chosen based upon the size of its cells and the accessibility of the embryos for experimental manipulation. Despite many advantages, the leech has all but vanished as a model system due, ironically, to its lack of genetic manipulability. Instead, as Ray anticipated, many neuroscientists focused on “genetic” model organisms that facilitated combined molecular, anatomical and physiological analyses in the context of a neural system.

Although Stent equivocated in his title, his intent was to question the ultimate utility—and to emphasize perceived weaknesses—of genetics in assessing the complexity of developing neural systems. In contrast, Ray's work demonstrated that there were only strengths in this approach to brain development and function—when used and interpreted appropriately. Ray showed that “anatomical” analysis—the cell biological foundation of modern systems neuroscience—in the context of single gene variation defines an important interface between the genome and an organism's ability to negotiate the environment. Ray knew that understanding this interface represented an important direction for neuroscience. Many of his trainees, including me, have spent their entire careers demonstrating the transcendence of Ray's foundational insights. Indeed, as summarized below, the strengths of the genetic approach combined with careful neuroanatomical analysis as pioneered by Ray, guided my efforts to understand the initial development of another neural system, the mammalian olfactory system.

## Same Approach, Different System

In my independent career, after my pre‐ and postdoctoral years, I set out on a potentially chimerical effort to use the new genetic approaches in mice to understand the early development of the mammalian forebrain, and how this information might provide a framework for understanding establishment of sensory systems. My approach reflected Ray's influence on my fundamental understanding of neuroscience, and his use of genetic and developmental observation to understand visual system structure and function. I focused, however, on a different sensory system: the olfactory system. This reflected my postdoctoral research analyzing postnatal circuit development in the primary olfactory pathway in mice. I recognized that the primary olfactory relay—afferents from olfactory receptor sensory neurons (ORNs) in the olfactory placode (OP; later the olfactory epithelium, OE) that coalesce into an olfactory nerve (ON), enter the olfactory bulb (OB) eventually form modular circuits via their connections within OB glomeruli—might also rely on sensory afferent mediated interactions for morphogenesis and early circuit differentiation. In fact, I specifically thought that afferents might influence subsequent olfactory development, as suggested by some early neuroembryological observations by Graziadei in the frog (reviewed by Dryer & Graziadei, 1994). The mystery I wished to solve, however, included how early molecular events, which I thought must rely on cell to cell signaling by a newly identified set of “inductive signaling” molecules and their receptors (Giguere, Ong, Segui, & Evans, 1987; Green & Smith, 1991; Thaller & Eichele, 1987), specified the olfactory pathway from the periphery to key central targets in the nascent forebrain.

**Figure 2:**
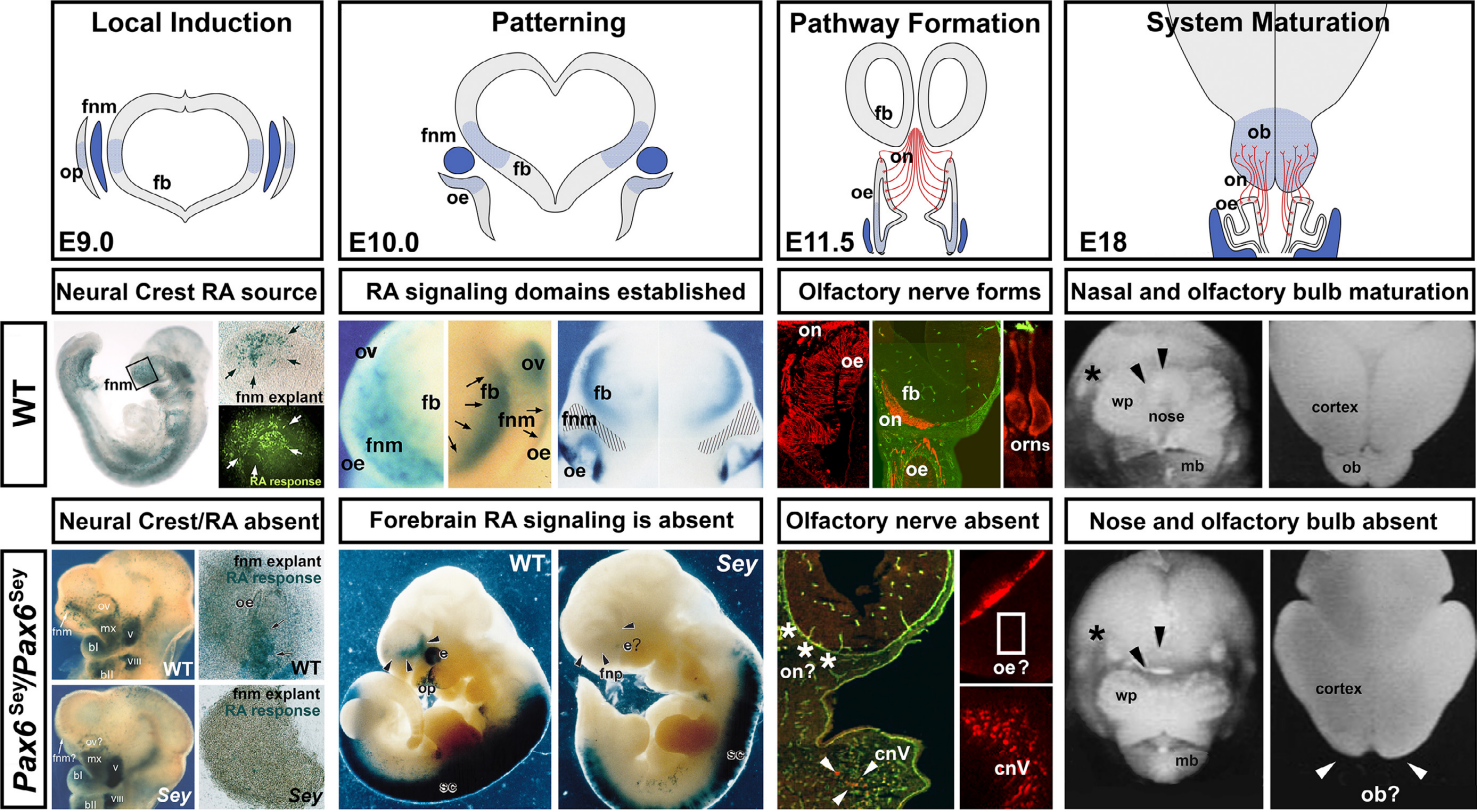
Using genetics to understand olfactory system development. Top row: (*left to right*): Induction, patterning, and neurogenesis establish the embryonic rudiments that subsequently form the primary pathway from the olfactory epithelium (oe) to the nascent olfactory bulb (ob). Growth and subsequent maturation of this pathway results in maturation and onset of normal sensory function in the olfactory system. Middle row: To accomplish the mechanistic steps necessary for olfactory system development in wild‐type (WT) embryos, a source of the inductive signal retinoic acid (RA; indicated in blue in the schematic) is established in neural crest‐derived mesenchyme between the cranial surface ectoderm and forebrain neuroectoderm (*far left panel*). E9.0 embryos express a reporter transgene that marks neural crest cells (*left*), and an *in vitro* assay for RA production (*right*) shows that these cells (explant, blue) are the RA source that selectively elicits an RA transcriptional response in an underlying monolayer of RA‐sensitive indicator cells (green). This local source (shaded area) patterns the ventral forebrain (fb), olfactory placode/epithelium (op, oe) and optic vesicle (ov) by E10.5 (*middle left panel*). These fb and oe RA‐mediated signaling domains are sites of early neurogenesis, and the olfactory nerve (on) is the first cranial nerve to reach its presumptive target (*middle right panel*). Subsequently (by E18.5), the oe and ob differentiate (*far right panel*) Bottom Row: In the *Pax6* mutant mouse *Small eye* (*Sey*), neural crest migration to the frontonasal mass (fnm) fails, and the mesenchyme no longer produces RA (*far left panel*). Failure of RA induction leads to failed fb and oe patterning (*middle left panel*), neuronal differentiation, on generation (*middle right panel*), oe and ob differentiation (*far right panel*; from: Anchan et al., 2000; Balmer & LaMantia, 2013; LaMantia, 1999; LaMantia et al., 1993, 2000).

I initially focused on local inductive interactions: signaling between distinct adjacent embryonic tissues that elicit changes in patterned gene expression and subsequent differentiation (Gilbert & Saxén, 1993). I believed that the molecular and genetic basis of these interactions in the forebrain might parallel those described in the spinal cord and hindbrain as well the limb bud (Smith et al., 1989; Tickle, 1991; Yamada, Placzek, Tanaka, Dodd, & Jessell, 1991). In the spinal cord, local inductive signaling mediated by distinct local sources apparently provided a framework for building circuits for motor control, and I assumed that there may be parallel functions for induction in the forebrain, particularly the primary olfactory pathway. There was, however, one caveat to studying such events in the forebrain: The structures that provide key inductive signals in the spinal cord: the notochord, floorplate, roofplate and somites were nowhere to be seen in or around embryonic regions that give rise to the forebrain (Figure 2, top row). Only three tissues were available: the anterior neural plate/tube, anterior cranial surface ectoderm, and a few mesenchymal cells in between.

The mechanistic contribution of each of these three embryonic tissues to forebrain differentiation and olfactory pathway development was defined using genetics—starting with a transgenic approach in mice that allowed visualization of molecular signaling initiated by Retinoic Acid (RA), one of the few embryonic inductive signals—at the time—known to operate both in the nervous system: the spinal cord and hindbrain, as well as in the periphery: limb buds and branchial arches (Colbert, Linney, & LaMantia, 1993; Marshall et al., 1992; Wagner, Han, & Jessell, 1992). With my colleagues Elwood Linney and Melissa Colbert, I showed that the ventrolateral portion of the early forebrain neuroepithelium, which gives rise to the olfactory bulb and ganglionic eminences (see below), is a target of selective RA‐mediated inductive patterning (LaMantia, Colbert, & Linney, 1993; Figure 2, top & middle rows). Surprisingly, there was a parallel domain of RA‐signaling in the adjacent surface ectoderm that includes the presumptive olfactory placode. These domains are the sites of earliest neurogenesis in the head and forebrain. At the time, it had been recognized that local sources of inductive signals mediate spinal cord motor neuron precursor patterning and neuroblast specification (Placzek, Yamada, Tessier‐Lavigne, Jessell, & Dodd, 1991). Accordingly, we reasoned that if RA‐mediated OP/ventral forebrain patterning represented true inductive signaling, there would be a distinct source for the RA signal that acted in a limited fashion to define these two domains. In a series of combined molecular and embryological experiments in vivo and in vitro, Melissa, Elwood and I demonstrated that RA was indeed available from local sources in the prevascular embryo (Colbert et al., 1993; LaMantia et al., 1993; Rubin & LaMantia, 1999). In the forebrain, this source was a bit of a surprise—RA was produced solely by the mesenchyme between the anterior surface ectoderm and the ventral forebrain neuroepithelium (Figure 2, middle row). Parallel work in the developing spinal cord (Colbert, Rubin, Linney, & LaMantia, 1995; Rubin & LaMantia, 1999), branchial and aortic arches, as well as limb buds (Bhasin, Maynard, Gallagher, & LaMantia, 1978), suggested that local RA sources acting upon a much broader distribution of RA receptors is a general feature of RA‐mediated induction in the mouse embryo (at least through E 11.5). Thus, prior to extensive vascularization of the embryo, RA is provided by specialized populations of cells at discrete sites to elicit RA‐dependent changes in gene expression via local activation of a limited subset of receptors in adjacent target tissues. These observations, made using genetic tools, provided a foundation for further analysis of olfactory pathway development.

### Olfactory development: Using genetics to understand the early assembly of a system

Ray's work—and his direct input—inspired me to consider how my initial observations might lead to a more detailed understanding of the development of the olfactory pathway. In 1993, as the final galleys for my first paper on these results “Retinoic Acid Induction and Regional Differentiation Prefigure Differentiation of the Mammalian Olfactory Pathway,” (LaMantia et al., 1993) were in hand, Ray visited Duke, where I was an assistant professor at the time. For reasons that were quite complicated, involving delayed flights and Duke Basketball, Ray ended up staying at my little house in northern Durham rather than the more palatial accommodations that had been planned. On the last day of his visit, I gave him the galleys of the paper to read, and nervously awaited his verdict. Once he finished, he said “I know what you're doing here, and I think you're going in the right direction.” I asked whether he thought I might move forward by looking for mutations in mouse that disrupted forebrain morphogenesis. He said that it might be helpful, but only if the mutation made sense in terms of its effect on the olfactory system—if that was my focus. With Ray's advice, validation and a focus on the olfactory *system* firmly in mind, I set out to find such mutations in mouse.

Two mutations emerged as likely candidates. The *Pax6* null mouse “Small Eye” (*Sey*/*Sey*) lacked an OE, ON and OB from the earliest stages of cranial/forebrain differentiation, in addition to better‐known eye anomalies seen somewhat later (Grindley, Davidson, & Hill, 1995). The *Gli3* null mutant “Extra Toes (Jackson)” mutant (*Xt* ^*J*^ */Xt* ^*J*^; Maynard, Jain, Balmer, & LaMantia, 2002) also lacked an OB; however, it had an OE with fully differentiated ORNs (Sullivan, Bohm, Ressler, Horowitz, & Buck, 1995) that gave rise to an ON that failed to enter the forebrain (Hui & Joyner, 1993). My choice of these two mutations, based upon olfactory *pathway* phenotypes, became a source of some controversy as I worked through these studies, due to the purported singular “function” of each gene: Pax6: the was the “master” regulator for eye development (Baker, 2005; Pichaud & Desplan, 2002), and *Gli3* a major focus in understanding *Shh*‐mediated signaling (reviewed by Villavicencio, Walterhouse, & Iannaccone, 2000). We ultimately resolved the contribution of mutations in *Pax6*,* Gli3*,* Shh*,* Raldh2* (the key synthetic enzyme for RA) and *Fgf8* for the differentiation of the forebrain, OE, ON and OB (Anchan, Drake, Gerwe, Haines, & LaMantia, 2000; Balmer & LaMantia, 2013; LaMantia, 1999; LaMantia, Bhasin, Rhodes, & Heemskerk, 2000; Maynard et al., 2013; Tucker, Polleux, & LaMantia, 2006; Tucker et al., 2008). Following Ray's example of careful anatomy in the context of single genetic mutations, we demonstrated that these mutations lead to altered OE neuronal differentiation, ON axon growth and OB morphogenesis (Figure 2, bottom row; Balmer & LaMantia, 2013; Bhasin et al., 1978; Haskell, Maynard, Shatzmiller, & LaMantia, 2002; LaMantia et al., 2000; Sherman et al., 2017; Tucker et al., 2006, 2008, 2010).

**Figure 3:**
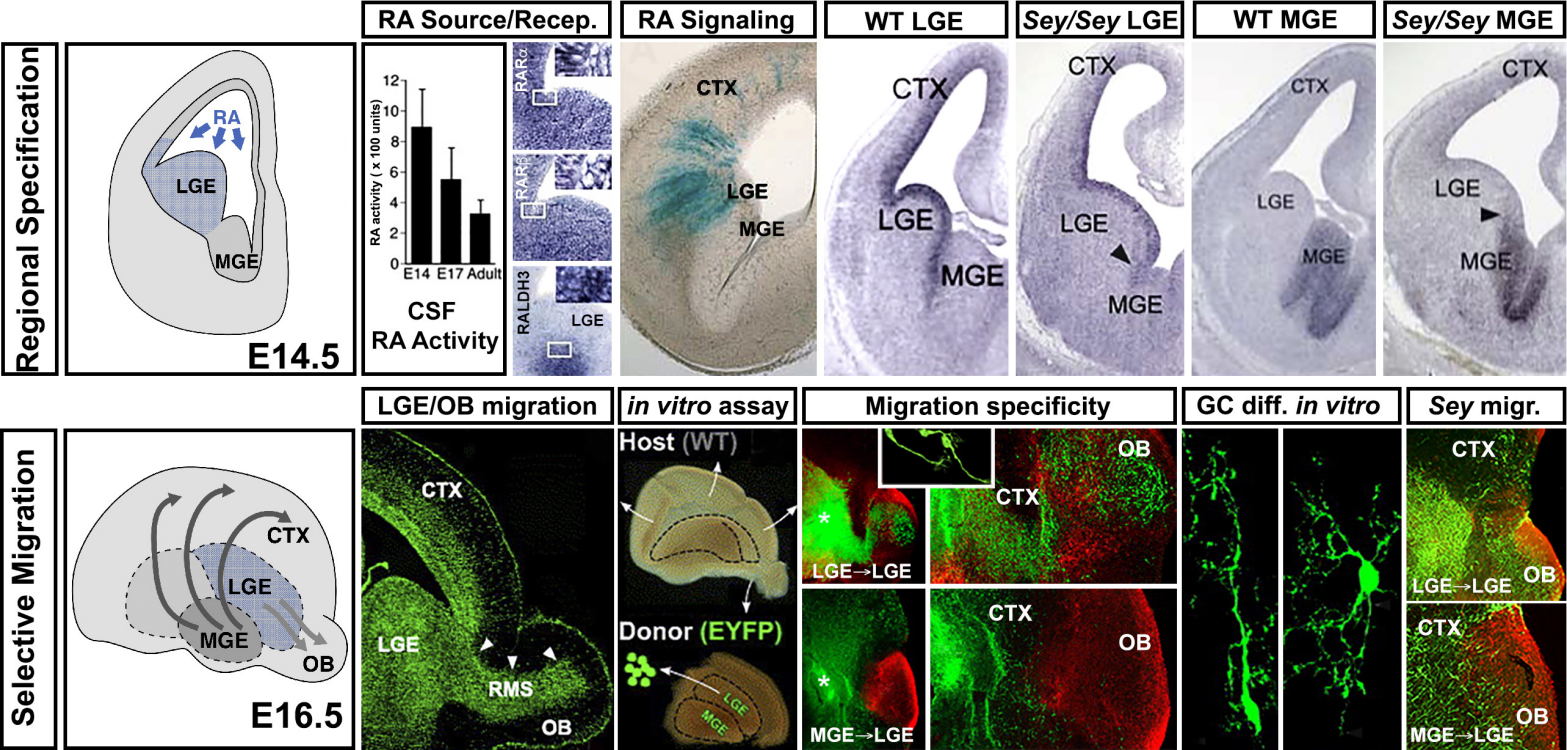
Genetic and cellular analysis of olfactory bulb morphogenesis. Top, *left panels*: A schematic of the E14.5 anterior forebrain, in coronal section, showing the lateral ganglionic eminence (LGE), source of OB interneurons, medial ganglionic eminence (MGE) which is the source of neocortical (CTX) interneurons. The blue shading indicates the RA‐signaling domain seen in the anterior forebrain at this age (LGE and part of CTX). RA is provided by the cerebrospinal fluid (CSF) within the lateral ventricle, acts upon RA receptors *RAR*α and *RAR*β, and is further metabolized by *Raldh3*, a RA synthetic enzyme, localized to the LGE. *middle right panels*: The E14.5 LGE/CTX and MGE are sites of limited gene expression, with LGE/CTX‐specific genes in register with the RA‐signaling domain. In *Pax6* ^Sey/Sey^, where the OB does not differentiate, LGE/CTX and MGE expression domains overlap, and expression levels appear diminished. Bottom row, *far left panel*: Specific migration of GABAergic LGE versus MGE generated interneurons. The *middle left panel* shows the distribution of E14.5 BrdU birthdated cells in an E16.5 forebrain sagittal section. The extension of LGE cells into the OB defines the rudimentary rostral migratory stream (RMS). The *middle panels* show an *in vitro* assay for LGE‐OB interneuron migration. A living forebrain hemisphere, dissected so that all domains (LGE, MGE, CTX, OB) remain, is placed flat on a tissue culture membrane. Donor EYFP LGE or MGE tissue is grafted into the LGE, and migration is analyzed after 4 days in vitro. The assay recapitulates migratory activity and specificity of LGE versus MGE cells: Only LGE EYFP^+^ cells migrate into the OB rudiment when placed in the host LGE. *middle right panel*: Differentiated LGE cells in the *in vitro* OB resemble granule (GC) and periglomerular (PG) cells. *far right panel*: The migratory specificity of *Pax6* ^Sey/Sey^ LGE versus MGE cells is diminished; both populations are seen migrating from the LGE to the OB as well as CTX after grafting into a WT host explant.

This genetic approach helped define the mechanisms that underlie OB differentiation (Figure 3). As OB morphogenesis proceeds (E14.5 onward), RA signaling continues to distinguish the ventral forebrain region that gives rise to the majority of OB neurons: the lateral ganglionic eminence (LGE; Haskell & LaMantia, 2005; Figure 3, top row, *left*). RA is now provided by the cerebrospinal fluid as well as perhaps synthesized locally in the LGE (Haskell & LaMantia, 2005; Lehtinen et al., 2011; Wagner et al., 1992 Figure 3, top row, left). The LGE generates OB interneurons that migrate specifically and in large numbers via the rostral migratory stream (RMS; Figure 3, bottom, row left) to drive OB morphogenesis. In mutants where OB morphogenesis fails (*Pax6* ^Sey/Sey^ shown here), patterning of LGE‐restricted genes is modified: These genes are no longer restricted to the LGE, their expression levels appear diminished, and perhaps counter‐intuitively, the LGE is expanded in size at the expense of the medial ganglionic eminence (MGE) the source of interneurons that migrate to the neocortex (CTX). This suggests that early patterning specifies LGE versus MGE progenitors to generate interneurons capable of migrating to the OB versus CTX. This is indeed the case: When explanted homologously (e.g., LGE to LGE) versus heterologously (e.g., MGE to LGE) using an in vitro assay, only LGE cells migrate into the rudimentary OB (Tucker et al., 2006; Figure 3; bottom row, left). These LGE cells differentiate into apparent OB granule and periglomerular cells in vitro, consistent with their presumed specificity. The consequence of this patterning and specification is somewhat surprising: They seem to disrupt the LGE versus MGE migratory selectivity. Thus, when placed in the LGE of a WT forebrain, *Pax6* ^*S*ey/Sey^ LGE and MGE cells migrate promiscuously, populating both the OB and the CTX. We suggest that the failure of LGE migratory specification of OB interneurons, plus the disrupted overall patterning of the anterior forebrain due to Pax6 loss of function (reviewed by O'Leary, Chou, & Sahara, 2007) combines to prevent the normal morphogenesis of the OB, even though some cell types may remain and constitute a remnant of OB‐like tissue at the anterior pole of the cortical hemisphere in *Pax6* ^Sey/Sey^ mutants (Jiménez et al., 2000).

Our goal was not to define the molecular function of Pax6, Gli3, Shh or Fgf8—that was being done by others (*Pax6*: reviewed by Cvekl & Callaerts, 2017; Hanson, 2001; Strachan & Read, 1994; *Gli3*: reviewed by Biesecker, 2006; Villavicencio et al., 2000; *Fgf8*: reviewed by Goetz & Mohammadi, 2013; Krejci, Prochazkova, Bryja, Kozubik, & Wilcox, 2009). Instead, we used these mutations, as Ray used the large series of albino mutations, as natural experiments. In our case, we integrated genetics and neuroanatomy to understand the initial steps of development of the olfactory system and forebrain. Ray recognized that the pigment mutations altered the qualitative and quantitative balance of retinal inputs to central visual targets. Similarly, my colleagues and I set off to identify a series of mutations that eliminated or dramatically disrupted the early differentiation of key peripheral or central structures in the primary olfactory pathway. Our work on modulation of RA signaling by these and other mutations (see below) outlined a mechanism for forebrain and olfactory pathway induction and early morphogenesis. This novel mechanism—no notochord or floorplate necessary—seemed remarkably similar to that for the primordia of the limbs, heart and face (Bhasin et al., 1978; LaMantia, 1999). At each location, axial coordinates are defined by localized sources of RA, Shh, Fgfs and Bmp—all of which rely on migration and initial differentiation of neural crest‐derived mesenchyme at each site, and these coordinates guide differentiation. Once our initial work on induction was complete, we showed that induction also influenced growth of OE axons into the OB (Balmer & LaMantia, 2013; Rawson et al., 2010; Tucker et al., 2010; Whitesides, Hall, Anchan, & LaMantia, 1998). We also established the capacity of neural crest mesenchyme to pattern additional cardinal signaling molecules (Fgfs, Shh, Bmps) in the OP and ventral forebrain (Bhasin et al., 1978; Tucker et al., 2008) to constrain afferent growth and further target differentiation, and the specificity of frontonasal versus branchial arch or limb mesenchyme for OE and ON differentiation (Rawson et al., 2010). This work relied upon principles derived directly from Ray's work in mink and other albinos: start with a primary change, and then evaluate its consequences in additional, relevant parts of the developing or mature nervous system.

### Noses, bulbs, faces, hearts and limbs: Different endpoints, a common mechanism

A novel hypothesis emerged from this exploration of induction and its influence on olfactory system development: The signaling mechanisms for limb, aortic arch and craniofacial morphogenesis were also used to build the forebrain—particularly to specify the OE, ON and OB (Bhasin et al., 1978; LaMantia, 1999). This relationship was discerned using relevant mutations combined with embryological and neuroanatomical observation—Ray's synthesis applied to a different sensory system. Using mutations that disrupted olfactory system development, as Ray did for visual system development, we found that the olfactory pathway relied critically on inductive interactions parallel to those used to build limbs, hearts and faces (Balmer & LaMantia, 2011; LaMantia, 1999, 2014; Maynard, Haskell, Peters, Lieberman, & LaMantia, 2003). Accordingly, mutations that disrupt olfactory pathway differentiation—and limb, face and heart morphogenesis—also disrupt local RA induction (Figure 2, bottom row). These disruptions prefigure the failure of olfactory pathway development (Balmer & LaMantia, 2011; LaMantia, 1999, 2014).

These studies in the olfactory pathway mirrored Ray's genetic approach in the developing visual pathway: Single mutations served as a natural experiment to understand the development of the *system*. They provided a foundation for establishing a molecular understanding of how the olfactory pathway differentiates as a coordinated system during the earliest phases of forebrain development. Once again, our results in the olfactory system paralleled those from Ray and his trainees into the consequences of pigment mutations for visual system organization. Ray's attention focused on the systemic consequences of the mutations and how the developmental mechanisms gave insight into fundamental visual system organization and function. He left it to his trainees, particularly Carol Mason and her colleagues (Herrera et al., 2003; Petros, Rebsam, & Mason, 2008) to elucidate the detailed molecular mechanisms. They found that a primary disruption of adhesion signaling between transcriptionally mis‐specified retinal ganglion cells and a distinct population of pigmented cells in the nascent optic chiasm is responsible for the subsequent disruption of visual pathway development in albino animals. Similarly, in the olfactory system, we found a singular molecular mechanism, induction via RA‐mediated M/E interactions, has concatenated effects that from the periphery onward facilitates differentiation of each olfactory pathway component (Figure 2; reviewed by Balmer & LaMantia, 2011; LaMantia, 2014).

### Following Ray's lead: genetic tools for insights in neural circuit development

In the early 2000s, as this work reached its midpoint, and my confidence in the genetic analysis of olfactory pathway induction and assembly as well as its contribution to forebrain differentiation grew, I began to wonder whether I could scan the literature for additional mutations that compromised brain, face, heart and limb development. These mutations would provide additional natural experiments to understand the apparently shared morphogenetic mechanism, as well as how this mechanism is modified in the forebrain to achieve a distinct, and arguably far more complicated, endpoint. I thought of this in the way that Ray's analysis of Siamese cats, then mink, mice, monkeys, tigers and even human albinos gave increasing insight into how the retino‐geniculo‐cortical pathway developed and worked. Thus, following Ray's lead, I decided to look for additional mutations that changed early forebrain development, including olfactory pathway differentiation and function.

I was not only inspired by Ray's foundational synthesis of genetics and neural systems in a range of mammalian pigment mutants, but by its further development by one of Ray's finest students, Chris Walsh. Chris adapted Ray's neuro‐genetic approach to understand human cerebral cortical development (reviewed by Hu, Chahrour, & Walsh, 2014; Walsh & Engle, 2010). With these dual inspirations, I looked for human mutations that had olfactory pathway dysmorphology (exceedingly rare) or limb, heart and face phenotypes (far more common). With encouragement from my colleagues Jeff Lieberman and Lynn Sikich at UNC‐Chapel Hill, I became fascinated by a multigene heterozygous microdeletion on human chromosome 22 and its associated phenotypes. In the late 1980s this deletion had been identified as the primary cause of DiGeorge syndrome (later renamed 22q11.2 deletion syndrome; McDonald‐McGinn et al., 2015), a developmental disorder in which children have limb and craniofacial anomalies, clinically significant heart malformations *and* a remarkably high incidence of social/cognitive behavioral disorders—which I interpreted, broadly, as evidence of disrupted forebrain development. These “forebrain” disorders were first identified as schizophrenia (Bassett et al., 1998; Karayiorgou et al., 1995), and later reclassified to include autistic spectrum disorders, attention‐deficit/hyperactivity disorder, depression and anxiety (reviewed McDonald‐McGinn et al., 2015; Meechan, Maynard, Fernandez, Karpinski‐Oakley, & LaMantia, 2015; Meechan, Rutz et al., 2015).

**Figure 4:**
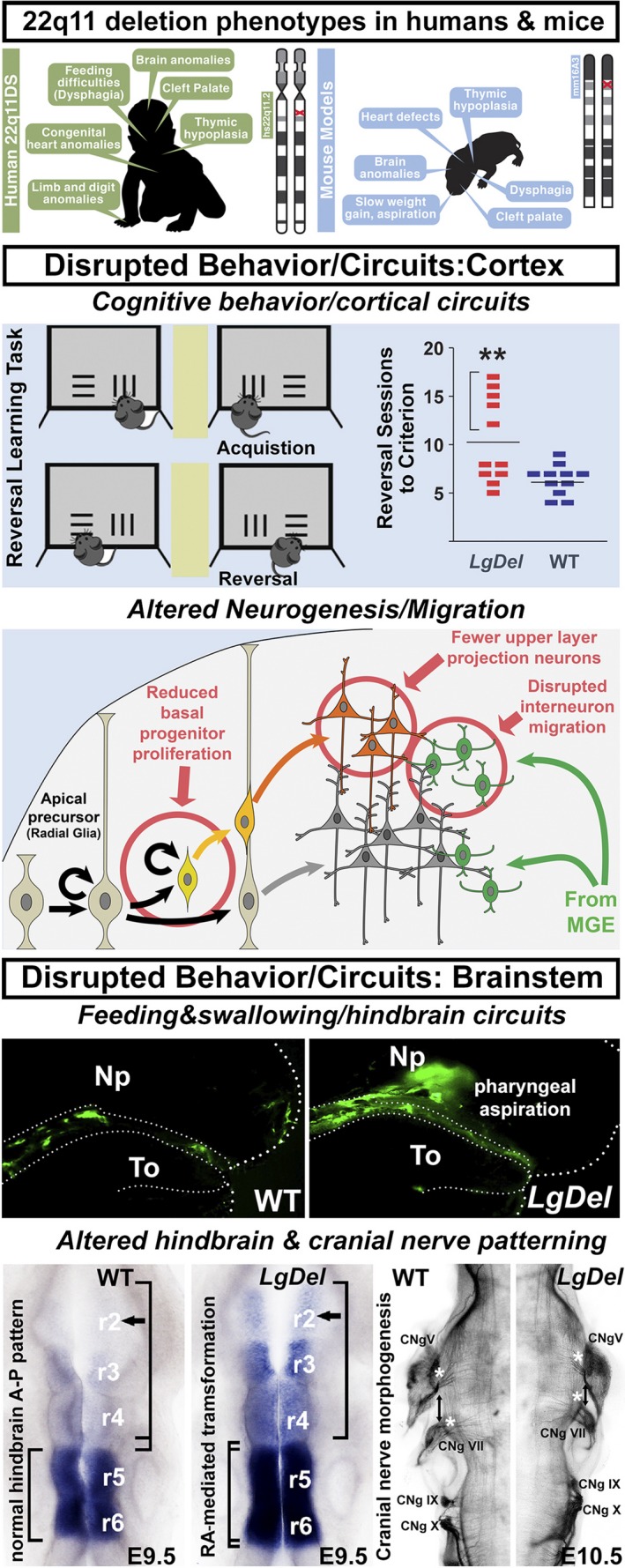
A human genomic “lesion” as a starting point to understand development and organization of complex behaviors and neural circuits. Top: Phenotypes associated with heterozygous deletion of 32 contiguous genes on human chromosome 22 (*left panel*) in children with DiGeorge/22q11.2 deletion syndrome (22q11DS). Deletion of 28 contiguous orthologous genes on mouse chromosome 16 (*right panel*) results in parallel phenotypes that can be recognized during early development through adolescence and adulthood in the *LgDel* mouse model of 22q11DS. Middle: Key behaviors, and presumably the underlying neural circuits, are disrupted in the *LgDel* mouse. *LgDel* mice have a cognitive behavioral deficit. Reversal learning—the capacity to learn a visual discrimination, for example, horizontal versus vertical orientation to receive a food reward (tested in touch screen apparatus as shown) and then reverse the reward contingency—is disrupted. *LgDel* mice, as a group, require significantly more sessions to learn the new reward contingency (*upper panel*). In *LgDel* mice, cortical circuit development is disrupted due to a basal progenitor‐specific proliferative deficit that results in diminished frequency of layer 2/3 projection neurons (the primary progeny of basal progenitors in the subventricular zone—SVZ) in association with cortical regions. In parallel, disrupted migratory capacity of MGE‐derived interneurons results in altered placement of parvalbumin‐labeled GABAergic interneurons In *LgDel* mice, these interneurons are seen at high frequency in layer 2/3, and lower in layer 5/6, opposite to the WT distribution (*lower panel*). Bottom: Using an in vivo swallowing assay that provides fluorescently labeled milk to suckling pups, normal swallowing can be seen in WT animals (minimal fluorescent residue in the mouth, tongue and esophagus), while *LgDel* animals aspirate into naso‐pharyngeal structures (*upper panels*). This malfunction of hindbrain motor and cranial sensory circuitry is prefigured by a retinoic acid (RA)‐mediated “posteriorization” of the rhombomere segments (r2–4), shown here by in situ hybridization of an RA‐sensitive gene whose expression expands and intensifies in *LgDel* embryos (*left and center*). The “posteriorized” rhombomeres are those that give rise to the trigeminal cranial sensory ganglion and motor nerve (CNg V, upper asterisks). This results in altered CNg V morphogenesis, placement and size (*right*). Furthermore, the relationship between the facial nerve and associated geniculate ganglion (CNg VII, lower asterisks) is altered (compare arrows in WT and *LgDel*).

With my colleagues Tom Maynard and Dan Meechan, as well as others in my laboratory over the past nearly 20 years, I set out to understand how deletion and diminished *dosage* of the minimal number of 32 genes on human Chromosome 22 (Carlson et al., 1997) leads to the constellation of phenotypes associated with 22q11DS (Figure 4, top row). Initially, we reasoned that by studying mutations in individual genes in the region, we would identify common mechanisms that modulate limbs, aortic arches, branchial arches and olfactory/forebrain induction (LaMantia, 1999). The mouse genome had an orthologous set of contiguous genes on murine chromosome 16 (which combines orthologues of genes on both hChr 22 and 21; reviewed in Maynard, Haskell, Lieberman, & LaMantia, 2002). Thus, we could analyze expression and function of these genes in the mouse and potentially use mutations in a subset of the genes to continue to understand olfactory pathway differentiation as well as broader issues of forebrain development. Our first step, a functional genomic analysis (expression quantification, patterning, regional and cellular localization throughout the embryo and in the adult) yielded a surprise: 22 of the 28 murine orthologues of genes within the minimal critical deleted regions associated with 22q11DS were expressed first at sites of nonaxial mesenchymal/epithelial induction: the limbs, the aortic arches, the craniofacial primordial and the frontonasal mass/forebrain. In addition, all 22 of these orthologues continued to be expressed at varying levels and in distinct cell classes in the developing and adult brain and spinal cord (Maynard et al., 2003, 2008; Meechan, Maynard, Peters, & LaMantia, 2007; Meechan, Tucker, Maynard, & LaMantia, 2009). Subsequently, using a mouse mutant with a heterozygous deletion of 28 contiguous, orthologous genes on mouse chromosome 16, the *LgDel* mouse (Merscher et al., 2001), we found that heterozygous deletion of these genes in *LgDel* disrupts the normal “dynamic range” for inductive signaling by RA, Shh, Fgfs and Bmps. In *LgDel* embryos, the capacity of the embryo to regulate changes in concentration or activity of these signals due to additional genetic or pharmacological changes in the signaling pathways is diminished dramatically. Alterations that have no effect in wild type have substantial phenotypic consequences in *LgDel* embryos (Maynard et al., 2013).

Finally, we began exploring whether other complex forebrain/disease phenotypes in 22q11.2 DS patients—ASD, ADHD and Scz—might reflect altered dosage of 22q11 genes in distinct neurons and glia, and its consequence for cortical circuit development and function. Using *LgDel* and mutations in individual 22q11 genes including *Tbx1*,* Prodh1* and *Ranbp1* (Maynard et al., 2013; Meechan et al., 2007, 2009; Paronett et al., 2015), we gained further insight into how diminished dosage cortical circuit development (reviewed by Meechan, Maynard et al., 2015; Meechan, Rutz et al., 2015; Figure 4, bottom middle and bottom panels). Joined by our collaborator Larry Rothblat, we assessed how diminished 22q11 gene dosage compromises cortico‐cortical connectivity via layer 2/3 cortical projection neurons and interneurons, and how these changes contribute to disrupted cognitive behaviors in *LgDel* mice (Meechan et al., 2009; Meechan, Tucker, Maynard, & LaMantia, 2012; Meechan, Rutz et al., 2015; Paronett et al., 2015). In addition, with Sally Moody and David Mendelowitz, we characterized development and function of cranial sensory and brainstem motor circuits for feeding and swallowing (Karpinski et al., 2014; LaMantia et al., 2016; Wang, Bryan, LaMantia, & Mendelowitz, 2017). We related a 22q11 deletion‐dependent disruption of RA‐mediated hindbrain patterning (Karpinski et al., 2014; LaMantia et al., 2016; Figure 4, *bottom panel*) to pediatric dysphagia—feeding and swallowing difficulty—that complicates early life for children with 22q11.2 DS (Eicher et al., 2000), thus establishing the *LgDel* mouse as the first genetically defined animal model for perinatal dysphagia in a neurodevelopmental disorder (reviewed by LaMantia et al., 2016). This work reflects the example set by Ray, using mutations as a starting point to assess a broader network of neurobiological mechanisms, and thus define development, structure and function of neural circuits and systems.

On my last visit to see Ray in Oxford in 2011, I gave an informal seminar to the neuroscience group at Oxford on the 22q11 work, and Ray came. As we talked afterward, I told him that I was considering turning the focus of the laboratory to the consequences of 22q11 gene dosage on circuit development for essential behaviors. He endorsed that direction with his usual understated but welcomed approval: “You should do that—it's good work.” Now, seven years later, we still approach 22q11 deletion as a starting point to understand the differentiation of specific neural circuits, systems and their physiological and behavioral function. Thus, I continue to follow Ray's lead: his essential template using mutations and neuroanatomy to discern how brains develop, and how they work.

## “Lock Up”: Remembering Ray and Looking Forward

During my years in Ray's lab in Chicago, I usually worked in the laboratory area just outside of Ray's office. Ray would often leave for dinner with his family while many of us, including me, continued to work in the laboratory. I remember distinctly that Ray never said “Goodbye” or anything like that as he closed his office door and passed by the laboratory bench where I was working. Instead, he would look at me, smile slightly, and say “Lock up.” It is a good and useful way of ending—close the door on one experiment, 1 day, one era, anticipating those to come. Ray provided his own last “Lock up” in his final book, The brain as a tool [a Neuroscientist's account] (Guillery, 2017), an elegant, witty and compelling summary of his truly remarkable life, his transformative scientific contributions, and his creative, sophisticated, rigorously philosophical view of *Neuroscience* (italics mine) beyond the disciplines of anatomy, physiology, genetics and all the rest. Ray knew that these disciplines often provide incisive and elegant methods but not integrated insight. During my time in Ray's lab in Chicago, I always tried to follow Ray's instruction: If I was the last to leave the laboratory in the basement of Abbot Hall, I always locked the door. We now all have a larger set of instructions to follow, left for us by Ray, extraordinary scientist, scholar, mentor and friend. We have to “Lock up” the door that Ray provided us, and open others to which he has given us access. Ray's remarkable synthesis of genetics and neuroscience provides direction for moving forward in this ongoing effort to understand how sensory systems—and indeed all neural systems—develop and function, and how they allow us to comprehend the world.

## Conflict of interest

The author has no conflicts of interest to disclose.

## Author contribution

The sole author of this article, Anthony‐S. LaMantia, wrote all of the text, assembled the figures and wrote the figure legends. The summary drawings in the figures were provided byThomas M. Maynard.
