# Supplementary material for: The strengths of the genetic approach to understanding neural systems development and function: Ray Guillery's synthesis
Source: Eur J Neurosci. 2018 Aug 1;49(7):888–99. doi: 10.1111/ejn.13985 (PMC6369024; doi:10.1111/ejn.13985)
Supplement: Supplementary file 1 [file EJN-49-888-s001.pdf]

EJN European Journal of Neuroscience FENS Federation of European Neuroscience Societies

The Strengths of the Genetic Approach to Understanding Neural Systems Development and Function: Ray Guillery's Synthesis
Anthony LaMantia

____

Review timeline: Submission date: 11.02.18
Editorial Decision: Minor Revision
Revision Received: 10.03.18
Editorial Decision: Minor Revision
Revision Received: 04.05.18
Accepted: 23.05.2018

Editor: Paul Bolam
Reviewer 1: Molnar Zoltan, University of Oxford, Department of Physiology, Anatomy and Genetics, United Kingdom

____

1st Editorial Decision 10.02.2018

____

The Strengths of the Genetic Approach to Understanding Neural Systems Development and Function: Ray Guillery's Synthesis

Dear Anthony,

Your manuscript has been reviewed by an external reviewer, the Editorial team as well as by Carol and Murray.

As you can see, the external reviewer really likes your review (as do Carol, Murray and ourselves) and considers it a great contribution to this Special Issue in honour of Ray. He/she presents a list of suggested revised which may improve/clarify part of the paper. Please carefully consider each point and revise accordingly.

EJN European Journal of Neuroscience FENS Federation of European Neuroscience Societies

We also noted the following points that need to be addressed in the revised version.
- End of page 21: it looks like there is a citation missing (empty set of brackets).
- Abbreviation list needed and some abbreviations not defined at their first use.
- Superscript numbers, asterisks and 'co-first author' need to be removed from the reference list.
- Higher resolution figures will be needed for publication.
- The figures need scale bars
- Please indicate the origin of the figures and ensure that permission if gained to reproduce them.
- Include USA in the address.

Thank you for your support of this Special Issue of EJN.

Best wishes,

Paul & John
co-Editors in Chief, EJN

Reviews:

Reviewer: 1

Comments to the Author
The Strengths of the Genetic Approach to Understanding Neural Systems Development and Function: Ray Guillery's Synthesis

The review is extremely well written, it is a personal tribute to Ray Guillery with excellent science spanning 3-4 decades. I have little to criticize and it could be published as it is, but I have the following suggestions to consider.

1. The review is built around the genetic modification of two different sensory systems and the effects on their development. The two figures (Fig. 1 and 2) are extremely useful, but I would urge the author to introduce the similarities and differences at an earlier stage of the review. Guillery used the genetics to alter the input to a system, whereas in the olfactory system the mutations that the author examined were at various sectors of the system. It would be good to emphasize this difference in the analysis earlier.
Expand sentence: "we used mutations in these and other genes, as Ray used a large series of albino mutations, as natural experiments. In our case, we integrated genetics and neuroanatomy to understand the development of the olfactory system and forebrain."
It would be also good to move the sentence below a bit earlier in the review for the general reader: "Ray correctly determined that chiasmatic crossing of retinal axons was mediated by pigment-related

guidance signals and then had effects that concatenated throughout the visual system."

2. It would be good to give a few general references at the end of this sentence at the early part of the paper: "Ray's foundational influence on my career can be seen in my reliance on the integration of genetics and neuroanatomy to understand neural systems development. His pioneering work on retino-geniculo-cortical development, its disruption by albino mutations and the consequences of those genetic "lesions" for the assembly of a functional system
provided a template for my own work on olfactory system development."

3. Guillery followed the literature on the early development of the thalamocortical and corticothalamic development and his predictions on the relative timing of the retinogeniculate and geniculocortical projections were confirmed with carbocyanine dye tracing experiments.
Ray used to say that "retinal projections plug into circuits that are to some extend already partially formed." Then the spontaneous and subsequently the sensory driven activity begin to influence their arrangement that was accomplished based on autonomous mechanisms. He was very interested in the role of the reticular and perireticular nucleus during development and considered them as the "subplate of the thalamus". It would be good to describe some of these ideas from the classic TINS review with Mitrofanis (Mitrofanis, J., and Guillery, R. W. (1993). New views of the thalamic. reticular nucleus in the adult and the developing brain. Trends Neurosci. 16, 240–245.).

4. More recent work on the development of the corticothalamic pathways also confirmed Ray's original suggestions. It would be good to explain this and add a few pertinent references near this sentence: "Ray, Mark Ombrellaro, and I (Guillery et al, 1985b) went on to show that the remaining, still topographic geniculo-cortical and cortico-geniculate projections likely contribute to the preservation of rudimentary dLGN differentiation in the complete absence of retinal input." Moreover, it was recently demonstrated that the development of corticofugal projections from layers 6 and 5 are altered after monocular enucleation. Ray helped with this manuscript and was excited to see that the ingrowth of layer 6 was accelerated after enucleation and also, layer 5, that normally does not grow into dLGN, started to develop projections to dLGN after monocular enucleation.
The Regulation of Corticofugal Fiber Targeting by Retinal Inputs. Grant E, Hoerder-Suabedissen A, Molnár Z. Cereb Cortex. 2016 Mar;26(3):1336-1348.

5. Reference missing from page 21: "early life for children with 22q11.2 DS (Eicher et al, 2000), thus establishing the LgDel mouse as the first genetically defined animal model for perinatal dysphagia in a neurodevelopmental disorder ()."

The review is excellent. It is brilliantly written; it is a superb mixture of a personal tribute and exciting and thought provoking timeless science.

EJN European Journal of Neuroscience FENS Federation of European Neuroscience Societies

____

Authors' Response 13.04.2018

____

Dear Paul and John:

Thanks so much for your wise editorial handling of my manuscript for the special edition of EJN honoring Ray. Thanks also to the anonymous review for her/his insightful and helpful comments.

I have uploaded a word document of the final text with references and figure legends. I have also uploaded the final figures at appropriate resolution. Finally, I have provided a graphical abstract that conforms, I hope, to the requirements of the journal.

As for permissions, some of the figure panels are from published work (the top images in Figure 1, some of the panels in Figures 2, 3 and 4) and some are taken from "alternate" images from my files to avoid the problem of permissions. I can clarify as soon as you need the information. All of the drawings in this review were generated de novo, although they may include some similar information to schematics published in several previous data papers and reviews.

I'll look forward to seeing the galleys of the manuscript. I should know this, but does EJN still publish bound volumes on paper, or is the journal completely on line at this point?

Thanks again. I feel very honored to have been part of this effort to remember Ray in the pages of the journal he founded.

best
A.____

2nd Editorial Decision 13.04.2018

____

The Strengths of the Genetic Approach to Understanding Neural Systems Development and Function: Ray Guillery's Synthesis

Dear Anthony,

Many thanks for the thoughtful and careful revisions of your paper and, as you can see, the reviewer is

very happy with them. We are thus pleased to say that it will be accepted for publication in the Special Issue of EJN after dealing with a two small points. First, please supply a graphical abstract. The second is the thorny and tedious business of permissions to reproduce figures. It is a relatively easy thing to do in this day-and-age, but still tedious. I will check with the editorial office whether we can go ahead while this processes. We will then send it off to the production team.

Please get back to us as quickly as possible.

Thank you for your support of this Special Issue of EJN in memory of our old friend.

Best wishes,

Paul & John
co-Editors in Chief, EJN

Reviews:

Reviewer: 1

Comments to the Author
The author responded to all my suggestions and the paper is now ready for publication. I would like to complement the authors for his superb review. His manuscript is not only a very valuable scientific review with exciting and timeless science, but it also contains a very warm and personal account of his interactions with one of the giants of neuroscience.
